# Accuracy Analysis of Digital Models from Intraoral Scanners and 3D-Printed Casts in Children and Teenagers

**DOI:** 10.3390/children11091082

**Published:** 2024-09-03

**Authors:** Diego Serrano-Velasco, Andrea Martín-Vacas, Giovanni Giovannini, Marta Macarena Paz-Cortés, Juan Manuel Aragoneses

**Affiliations:** 1 PhD Program in Translational Medicine, Universidad San Pablo-CEU, CEU Universities, 28003 Madrid, Spain; polackdiego.serranovelasco@usp.ceu.es; 2 Faculty of Dentistry, Alfonso X El Sabio University, 28691 Madrid, Spain; giovanni@uax.es (G.G.); mpazcor@uax.es (M.M.P.-C.); jaraglam@uax.es (J.M.A.); 3 Department of Dental Research, Federico Henriquez y Carvajal University, Santo Domingo 10106, Dominican Republic

**Keywords:** dentistry, dimensional measurement accuracy, imaging, three dimensional, orthodontics, pediatric dentistry, printing

## Abstract

Purpose: The aim was to analyze the accuracy of digital models and 3D-printed casts from full-arch digital impressions using two intraoral scanners (iTero^TM^ and Primescan^TM^). Materials and methods: A crossover reliability study was designed, scanning children and teenagers with iTero^TM^ and Primescan^TM^. Accuracy was evaluated by measuring intercanine, intermolar, and ipsilateral canine–molar distances intraorally and comparing these measurements with those from plaster casts, digital models obtained with intraoral scanners, and 3D-printed casts. A paired comparison and a general linear model with a one-way repeated measures ANOVA procedure were carried out with a confidence level of 95% (*p* ≤ 0.05). Results: A total of 51 subjects were analyzed (mean age 12.35 ± 2.57). Statistical differences (*p* < 0.05) were found in the upper and lower arch regarding accuracy in comparison to intraoral measurements, except for the iTero^TM^-printed cast and canine–molar upper right and intercanine lower distances (*p* > 0.05 for all comparisons). Regarding a comparison between reproduction methods, the plaster cast oversized the intercanine upper distance in comparison with both intraoral scanners’ digital models and the Primescan^TM^-printed cast (*p* = 0.001), but there were no differences in the lower arch (*p* > 0.05 for all comparisons). Conclusion: Intraoral scanners reproduce tooth structures with similar accuracy to conventional methods.

## 1. Introduction

The concept of an intraoral scanner (IOS) was developed in the 1970s [1], and nowadays, multiple IOS systems are integrated into dental workflows. Among the advantages are real–time visualization, simplicity, image analysis options, rapid disinfection, and improved comfort [2,3,4,5]. However, there are some disadvantages [3], including a higher learning curve, the inability to modify the patient’s occlusion, and elevated costs.

The comparison between digital and conventional impressions for full-arch reproduction indicates that reliability is similar, with both techniques demonstrating high accuracy [6]. As a result, in recent years, intraoral scanners have become a common method of impression in dentistry. Various factors can affect accuracy, including scanning distance and angle [7], changes in environmental temperature [8], ambient light variations, scanning length [9], scanning technology, calibration, operator experience, scanning editing, head size [10], interdental spaces, arch length, restorations, implants, orthodontic appliances, surface characteristics [11], and crowding [12]. Furthermore, the accuracy of the maxillo–mandibular relationship registration may be affected by factors such as arch extension, edentulous spaces, or dental mobility [13].

Although the use of IOSs in children is a favorable choice, the evidence regarding their reliability and reproducibility is not strong due to differences in study methodologies [14,15,16,17,18,19,20]. However, the differences between intraoral measurements and digital models are clinically acceptable [21]. Furthermore, space analysis on digital models is just as reliable as analysis on plaster casts, with less variability between examiners [22]. Regarding the reliability of casts printed with 3D printers, it was concluded that the factor that most affects reliability is the type of 3D printer [23] and not the intraoral scanner. The printed casts have similar accuracy and trueness to plaster casts and are acceptable, although significant differences are observed between them.

The high prevalence of dental malocclusions in pediatric subjects justifies the necessity of using accurate instruments to reproduce dental structures. Factors such as the tooth replacement process, behavioral issues, and smaller oral cavities can also impact the performance of IOSs. Additionally, there are concerns about the ability of intraoral optical scanners to scan arches with partially erupted teeth, the alveoli of recently exfoliated teeth, or intermittent gaps resulting from the tooth replacement process. Furthermore, it should be noted that adolescents have arches with permanent teeth but still have reduced mouth opening compared to adult patients, which can complicate access. Although an in vitro study reveals that the size of the mouth opening in children does not affect the reliability of scanning [24], other authors have stated that accuracy is poorer in posterior areas compared to anterior ones, possibly due to the difficulty in accessing posterior teeth [20]. Therefore, reliability can be analyzed by comparing the plaster casts (gold standard) with digital models [20]. The aim was to analyze the accuracy of digital models and the 3D-printed cast from full-arch digital impressions using two IOSs (iTero^TM^ and Primescan^TM^).

## 2. Materials and Methods

A monocentric, controlled, crossover reliability study was designed according to the Guidelines for Reporting Reliability and Agreement Studies (GRRASs) statement [25]. This study was approved by the Ethics Committee of the San Carlos Clinical Hospital (code 21/336-E, 3 June 2021) and complied with the Declaration of Helsinki for biomedical research and current national and European regulations concerning personal data protection.

Due to the heterogeneity in previous studies, the sample size calculation was carried out in theoretical terms and not with previous data. The sample size was calculated using G*Power software (version 3.1.9.7., Düsseldorf, Germany) with an a priori procedure for the difference between two dependent means (matched pairs) [26,27]. In total, 34 subjects were needed with an alpha error (p) of 0.05, 80% power, and a medium effect size (0.5). To solve a possible loss of 50% of the subjects due to the collaboration, the initial sample size was increased to 51 subjects. Through the non-probabilistic sampling of consecutive cases, we invited subjects (1) under 18 years old who (2) requested an occlusion study at the clinic of the orthodontics master’s program at the Alfonso X El Sabio University (Madrid, Spain). We excluded (1) subjects with craniofacial syndromes, (2) systemic diseases, (3) behavioral problems, (4) intellectual disabilities, or (5) fixed appliances that could introduce biases in impression-taking/digital scanning. Written informed consent was obtained from the parents and/or legal guardians of the minors (under 16 years old), and acceptance was also requested from children over 12 years old in accordance with current regulations on the protection of personal data.

During the initial visit, an orthodontic evaluation was conducted, obtaining a conventional full-arch alginate impression for the preparation of plaster study casts and an intraoral scan using iTero Element^TM^ 5D Plus (iTero^©^ 2019 Align Technology, Inc., San José, CA, USA). All patients who met inclusion and exclusion criteria were scheduled for a second visit to be scanned with Primescan^TM^ (Primescan^TM^, Dentsply-Sirona^TM^, New York, NY, USA). Conventional alginate impressions (Orthoprint—Zhemarck SpA, Badia Polesine, BO, Italy) were taken using steel impression trays (Henry Schein Inc., Queens, NY, USA). The alginate was hand-mixed with tap water, and the impressions were poured within a maximum period of 60 min while being stored in a moist environment. IOSs (iTero Element^TM^ and Primescan^TM^) were performed according to the guidelines of each manufacturer, with the patient lying down and following the same sequence. The scanning and impression environment was stable, always taking place in the same facilities, with a similar ambient temperature (21–23 °C) and artificial light. The digital models were exported in Standard Tessellation Language (STL) format and stored on a multimedia hard drive, safeguarded by the project’s Principal Investigator (D.S.-V.). To blind the investigator, maintain patient anonymity, and protect their identities, the STL files were stored with the study patient numbers followed by the initials of each IOS.

The 3D-printed casts were printed from the STL files of both IOSs using a 3D Dental Microlay Versus^®^ printer (Microlay ©, Madrid, Spain) with Digital Light Processing (DLP^®^) technology. The printer specifications included a 12 V voltage, 180 W power, horizontal resolution of 65 µm, vertical resolution of 50 µm (up to 10 µm), accuracy of ±30 µm and Full HD LED projector with a wavelength of 385 nm (ultraviolet light) featuring an internal radiometer. All models were printed using a light-curing resin (Optiprint^®^ Sprint, Dentona, Dortmund, Germany), and calibrated according to the standardized printing protocol provided by the manufacturer. Model customizations were carried out using the Medit Model Builder app (version 1.3.3.70) integrated within Medit Link software (version 3.1.1. Build 261 Operation Europe, https://www.medit.com/). Printing settings were managed with MicroForm^®^ software (version 1.0.9.2, Microlay ©, Madrid, Spain).

**Figure 1:**
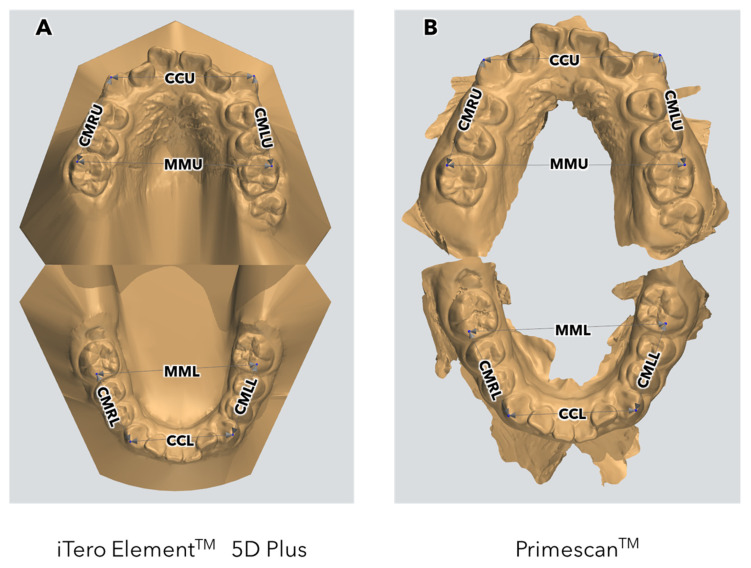
Intra-arch measurements for Subject #43 (10 years old) in digital models acquired with iTero ElementTM 5D Plus (**A**) and PrimescanTM (**B**) IOSs.

According to ISO 5725-1:2023 [28], the term accuracy in relation to a measurement method is defined in terms of trueness and precision. Trueness refers to the closeness or agreement between the arithmetic mean of a large number of test results and reality or the accepted reference value. Precision, on the other hand, refers to the closeness or agreement between test results obtained under stipulated conditions. Therefore, the measurement of accuracy was carried out through trueness, evaluating the digital methods in comparison to the real (intraoral measurements) or the gold standard (plaster casts). The following measures were carried out (Figure 1):

Intercanine distance: the distance between the cusp tips of the upper canines (CCU) or lower canines (CCL).

Intermolar distance: the distance between the mesio-buccal cusp tips of the upper (MMU) or lower (MML) first permanent molars.

Canine–molar distance: the distance between the cusp tips of the canine and the mesio-buccal cusp tips of the first permanent molars, measured in the upper right (CMRU) and left (CMLU) sides, as well as the lower right (CMRL) and left (CMLL) sides.

The measurements of intraoral, plaster casts, and 3D-printed casts were performed by the same operator (D.S.-V.) using the same electronic digital caliper (Vietnam E-Commerce Limited, RM 18 27/F, Ho King, COMM CTR 2 16 FA Yueng St. Mongkok, Kowloon, Hong Kong) (Technical Specifications in Appendix A). Digital models were measured by a second operator (G.G.) using Ortho Analyzer software (3Shape Ortho System 2022, 3Shape A/S, Copenhagen K, Denmark). To carry out the intraoral measurements directly with the electronic digital caliper, the subjects were placed in a supine position, and the oral cavity was illuminated with the light from the dental equipment. The measurements were carried out by introducing the caliper into the children’s mouths and previously explaining the procedure to them. To avoid eye strain, a maximum of five pairs of models or casts were measured per session, always in the same room with the same artificial light and closed blinds to prevent interference from natural light. Both operators (D.S.-V. and G.G) were previously calibrated.

Due to inherent differences in scanning or impression methods, neither the operator nor the patient could be blinded to the procedure. However, the measurements of the casts and models were performed blindly by two operators (D.S.-V. and G.G.). The data were tabulated and statistically analyzed by another researcher (A.M.-V.), blinded to the procedures, to avoid bias.

Intra-operator agreement analysis was conducted on a random 10% of the sample using the intraclass correlation coefficient (ICC), with a two-factor mixed model, absolute agreement procedure, and was interpreted according to Koo and Li [29]. Descriptive statistics were performed, and adjustment to a normal distribution was evaluated with the Kolmogorov–Smirnov and Shapiro–Wilk tests. A correlation analysis using the Pearson coefficient, interpreted according to Chan [30], was conducted to observe the relationship between the intraoral measurements and the reproduction methods. To analyze the accuracy of comparing the intraoral measurements, a paired test was conducted with Student’s T test or Wilcoxon’s rank test. To test the hypotheses of differences in the measurements across the three impression/scanning methods used, a general linear model was used, with one-way repeated measures (within subjects), the ANOVA procedure, and Greenhouse–Geisser (G-G) correction. The significance values of the post hoc tests for the pairwise comparison of variables were adjusted with the Bonferroni method. The tests were conducted using SPSS 24^®^ software (version 24.0, Armonk, NY, USA) with a confidence level of 95% (*p* ≤ 0.05) and asymptotic or bilateral significance.

## 3. Results

### 3.1. Sample Description

A total of 51 subjects were included in the study, with 19 in mixed dentition and 32 in permanent dentition. The mean age of the total sample was 12.35 ± 2.57 years (10.26 ± 1.85 in mixed dentition and 13.59 ± 2.09 in permanent dentition), with an age range of 7–17 years. The age distribution, both in the overall sample and within each dentition group, met the normality criteria (*p*-value > 0.05 for all cases).

### 3.2. Descriptive Statistics

The mean and standard deviation of all measurements were calculated (Table 1 and Table 2). The ICC results indicated good intra-operator agreement for the intercanine distance with Primescan^TM^ (ICC = 0.896) and excellent agreement for the rest of the measurements (ICC > 0.9 in all cases). In terms of the adjustment to normal distribution, all CCU, MMU, and CCL distances met the normality criteria (*p*-value > 0.05 in all cases). However, for the remaining variables, while some measurements met the normality criteria in certain reproduction methods, others did not, resulting in inconclusiveness.

### 3.3. Accuracy Analysis

Firstly, to observe how the measurements of the reproductions compared to the intraoral measurements, the Pearson correlation coefficient (Table 3) was carried out, obtaining statistically significant results in all cases (*p* < 0.001 for all comparisons). As can be seen, in the maxilla, the correlation was very high for iTero^TM^ STL-, Primescan^TM^ STL-, and Primescan^TM^-printed cast for all measurements. However, the results suggest that in the plaster cast, although the CCU and MMU measurements had a very high correlation, the correlation for CMRU was high, and for CMLU, it was low. On the other hand, in the case of the iTero^TM^-printed cast, all the correlations obtained were very high except for CMLU, which was high. In the case of the mandible, only the printed casts of the two intraoral scanners obtained very high correlations for all measurements. However, the results indicate that the correlation was high for the CMRL variable and moderate for the CMLL variable in the iTero^TM^ STL and Primescan^TM^ STL reproductions, respectively, although the rest of the measurements obtained a very high correlation. In the case of the plaster cast, although the CMRL and CMLL measurements obtained a very high correlation, it was low for the MML measurement and high for the CCL measurement.

Related to the analysis of reliability in the upper arch (Table 4), the most stable record compared to intraoral measurements was the iTero^TM^-printed cast, and the measurement less variable CMRU. In contrast, the iTero^TM^ STL and Primescan^TM^ STL showed significantly lower measurements in the CCU variable (with a mean difference of 0.409 mm for both variables) than the intraoral measurements. The MMU measurement resulted in a value significantly lower than intraoral distances in the Primescan^TM^ STL- and Primescan^TM^-printed cast (with a mean difference of 0.367 mm and 0.287 mm, respectively). The plaster cast significantly overestimated the CMLU measurement (with a mean difference of −1.329 mm) compared to the direct intraoral measurement.

Regarding the lower arch (Table 5), the most stable measurement was CCL, with no significant differences across any reproduction methods. The MML measurement was significantly lower in all reproduction methods except for the plaster model (with mean differences of 0.795 mm, 0.796 mm, 0.545 mm, and 0.542 mm, respectively). The CMRL measurement was significantly higher than the direct intraoral measurement in the plaster models and the printed models of iTero^TM^ and Primescan^TM^ (with mean differences of −0.569 mm, −0.458 mm, and −0.513 mm, respectively). The CMLL measurement was significantly higher in all cases (with mean differences of −0.508 mm, −0.030 mm, −0.668 mm, −0.374 mm, and −0.381 mm, respectively) compared to direct intraoral measurements.

### 3.4. Comparison between Reproduction Methods

**Figure 2:**
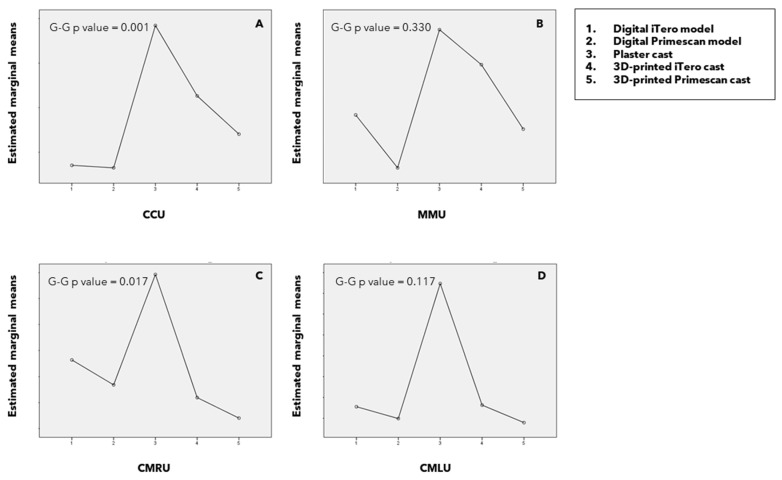
Profile graph illustrating intercanine (CCL) (**A**), intermolar (MML) (**B**), canine–molar right (CMRL) (**C**), and canine–molar left (CMLL) (**D**) distances in the upper arch. Intrasubject differences can be examined (G-G *p* values significant ≤ 0.05).

Differences between the reproduction methods were analyzed in the upper arch (Figure 2), yielding significant differences in the CCU measurement (G-G *p* = 0.001) and CMRU measurement (G-G *p* = 0.017). There were no significant differences in the MMU and CMLU measurements across the different reproduction methods (G-G *p* = 0.330 and *p* = 0.117, respectively). These differences were attributed to the plaster cast having significantly higher CCU measurements compared to iTero™ and Primescan™ digital models and Primescan™-printed casts. In the post hoc tests, no differences were found in the CMRU variable among the different reproduction methods.

**Figure 3:**
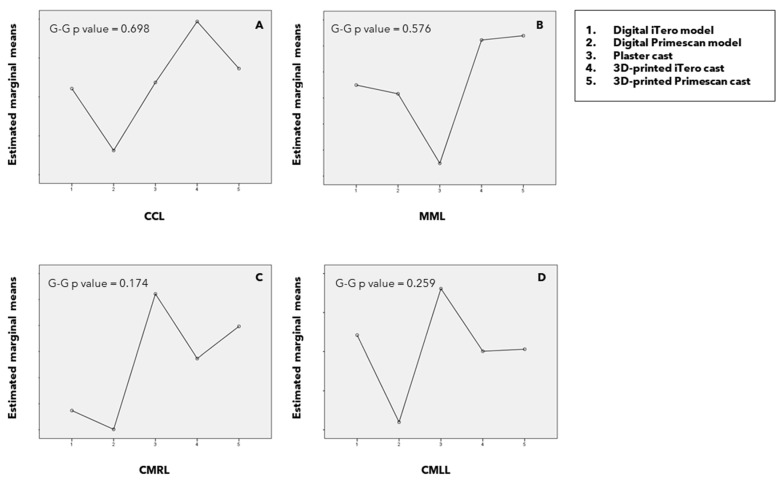
Profile graph illustrating intercanine (CCL) (**A**), intermolar (MML) (**B**), canine–molar right (CMRL) (**C**), and canine–molar left (CMLL) (**D**) distances in the lower arch. Intrasubject differences can be examined (G-G *p* values significant ≤0.05).

In the lower arch, all measurements (CCL, MML, CMRL, and CMLL) were similar across the different reproduction methods (Figure 3), with no statistically significant differences among them (G-G *p* = 0.698, *p* = 0.576, *p* = 0.174, and *p* = 0.259, respectively).

## 4. Discussion

IOSs have become a common element in dental clinics due to their numerous advantages over the conventional impression method. Systematic reviews on the reliability of IOSs [6,14,21] affirm the high reliability of digital full-arch reproduction methods, which are comparable to traditional impressions. However, the reliability of this digital procedure in children and teenagers has not been widely studied, leaving a gap in knowledge. Due to the distinctive characteristics of the pediatric population compared to adults, such as a smaller mouth size, varying levels of cooperation, and tooth replacement, this discussion focuses on studies conducted in children and teenagers.

There are only two studies that analyze reliability in children. Liczmansky et al. [20] examined 26 patients (with a mean age of 9.3 for boys and 9.8 years for girls) in mixed dentition, performing intraoral scanning with TRIOS^®^ Ortho (3Shape, Copenhagen, Denmark) and the desk scanning of plaster casts with the ATOS-SO^®^ system (GOM GmbH, Braunschweig, Germany). Three-dimensional differences were analyzed using GOM Inspect^®^ software (V2020). Glisic et al. [17] studied 59 subjects (with a mean age of 12.83 for girls and 12.56 years for boys), scanned with TRIOS^®^ Classic (3Shape, version 1.4.6.0., Copenhagen, Denmark). Intra-arch differences were compared (intermolar, intercanine, and ipsilateral canine–molar distances) measured with a digital caliper and 3Shape software (Ortho Systems 2015-1, version 1.6.1.10, Patch 10). In our study, we included 51 subjects (with a mean age of 12.35 ± 2.57 years), comparing intraoral measurements with plaster casts, digital models, and 3D-printed casts obtained from two IOSs, measuring with a digital caliper and Ortho Analyzer software. We considered plaster casts as the gold standard, similar to other authors [17,20]. Additionally, there is evidence that IOSs have an equal or lower precision than other impression materials, such as polyether or vinylsiloxanether [31].

The null hypothesis that the reliability or accuracy obtained with digital impression methods, as well as on 3D-printed casts, is comparable to conventional impression methods can be confirmed, with the results of this study. In the study by Liczmansky et al. [20] an absolute mean difference of 0.022 ± 0.027 mm was found, with no effects based on the stage of dentition, arch, or dental malocclusion. In the research by Glisic et al. [17], it was found that the intraoral distances were significantly smaller in the upper intercanine distance compared to the plaster casts (mean difference of −0.2 ± 0.54 mm) or the digital models (mean difference of −0.2 ± 0.51 mm), and in the distance between the upper right molar and canine compared to the plaster cast (mean difference of −0.28 ± 0.65 mm) and the digital model (mean difference of −0.36 ± 0.65 mm). However, no differences were found between the plaster casts and the digital models. According to our results, we found that the plaster casts significantly oversized the canine–molar left distance in the upper arch and both sides in the lower arch compared to intraoral measurements, with mean absolute differences between 0.569 and 1.329 mm. In addition, it appeared that measurements in the upper arch from digital models and printed casts were diminished in comparison to intraoral distances (mean absolute differences 0.287–0.409 mm), while they were augmented in the lower arch (mean absolute differences 0.030–0.796 mm). However, these differences were less than 1 mm and, therefore, clinically insignificant.

Although it is not possible to directly compare our results with those obtained in studies involving adults due to fundamental differences in oral and arch size, data derived from a meta-analysis [6] established that the maxillary distances and mandibular intercanine distance tended to be underestimated in digital methods, whereas mandibular intermolar distances tended to be overestimated, according to the obtained results. Furthermore, the 3D precision analysis showed a deviation of less than 0.1 mm in the overlap of repeated measurements, with greater deviations observed in the maxilla than in the mandible. It is challenging to establish a consistent pattern of errors in IOSs, but greater deviations are typically found in posterior regions [32,33,34,35], obtaining more precise results in the anterior area [36]. This results in a horizontal centrifugal expansion, which may be attributed to the increased difficulty of accessing posterior regions or errors in scanning strategy and/or in software processing [32,34,37]. According to previous authors, we found greater differences in the molar region in the lower arch, but this was also similar in the upper arch. Furthermore, as established in the published literature, it is possible to find positive or negative distortions when using intraoral scanners [36], as similarly observed in our results.

One possible limitation of intraoral scans in children is the size of the scanner heads, which can make accessing posterior regions difficult [20] and more limited cooperation from younger patients. Additionally, performing linear measurements on 3D structures introduces a bias when interpreting the data, as there may be differences in other spatial planes that are not analyzed. Therefore, it is essential to conduct further studies to corroborate and validate these findings. The exclusively theoretical calculation of the sample size without previous data could, on occasion, also underestimate the necessary size; however, the calculation of the post-analysis sensitivity offered positive data regarding this point, so it should be considered that the sample size used was adequate. Lastly, since the patients were in the dental replacement stage, not all variables could be analyzed in all cases, so the number of studied variables varied, although the minimum required sample size was always exceeded. The older age group consisted of teenagers who had already completed the exfoliation of their primary teeth but whose permanent teeth, although clinically erupted, had not yet completed the full eruption process to achieve complete intercuspation. The assessment of the intraoral scanners’ ability to reproduce arches with partially erupted teeth was also part of the original research question, and thus, the study design included this group of subjects. Therefore, while they were classified as having permanent dentition, it was not fully complete permanent dentition. This creates significant differences compared to adults, who present with fully complete permanent dentition and greater mouth opening. For this reason, we consider their study to be relevant, as they belong to the group of individuals who most frequently seek orthodontic evaluation.

On the other hand, the conducted research presents some strengths, analyzing two intraoral scanners and their printed casts in an abundant population requiring oral impressions, including children and teenagers. Among the strengths, we also found that the study of the current “gold standard”, plaster casts, is similar to the new reproduction methods. The use of new technologies is an advancement in dentistry, as it saves time and storage space. Therefore, the evidence of their comparable accuracy to conventional methods is a significant advantage. The comparison of modern digital dentistry techniques with conventional methods is a cutting-edge topic, with studies analyzing the validity of estimators such as the Bolton index, space analysis, or arch length, yielding promising results [38,39,40]. However, some authors still assert that plaster casts are more accurate [41]. Moreover, the initial investment required for an IOS takes approximately 3.5 years to break even compared to the conventional method [17].

As a future line of research, a study is proposed to evaluate a group of children and adolescents compared to a matched group of adults to observe if there are differences in reliability between population groups. It would also be relevant to analyze the reliability of the digital method in younger children with primary dentition; however, there are greater difficulties in recruiting this very young age group.

## 5. Conclusions

Digital models and 3D-printed casts from intraoral scanners reproduce tooth structures with similar reliability compared to the conventional method. Plaster casts tend to oversize some measurements compared to intraoral structures, while digital models and 3D-printed casts can oversize or undersize measurements in comparison to intraoral distances. However, these differences are considered clinically acceptable.

## Figures and Tables

**Table 1:** Measurements (mean and SD) and normality tests in the upper arch.

Upper Arch		Descriptive		Normality Tests	
		Mean (mm)	SD ^a^	Kolmogorov–Smirnov ^b^ *p* Value	Shapiro–Wilk *p* Value
Intercanine distance (CCU) (*n* = 41)	Intraoral (*n* = 45)	34.90	2.82	0.200	0.247
	iTero^TM^ STL (*n* = 43)	34.48	2.86	0.200	0.848
	Primescan^TM^ STL (*n* = 46)	34.36	2.91	0.200	0.816
	Plaster cast (*n* = 46)	35.02	2.61	0.200	0.996
	iTero^TM^-printed cast (*n* = 45)	34.74	2.93	0.200	0.920
	Primescan^TM^-printed cast (*n* = 46)	34.49	2.96	0.200	0.760
Intermolar distance (MMU) (*n* = 48)	Intraoral (*n* = 50)	51.84	2.96	0.200	0.890
	iTero^TM^ STL (*n* = 48)	51.65	3.12	0.186	0.416
	Primescan^TM^ STL (*n* = 50)	51.47	2.82	0.200	0.865
	Plaster cast (*n* = 50)	51.78	2.91	0.200	0.943
	iTero^TM^-printed cast (*n* = 50)	51.72	3.23	0.200	0.526
	Primescan^TM^-printed cast (*n* = 50)	51.55	2.84	0.200	0.599
Canine–molar distance (right) (CMRU) (*n* = 41)	Intraoral (*n* = 45)	22.91	2.06	0.200	0.299
	iTero^TM^ STL (*n* = 43)	23.08	1.75	0.200	0.124
	Primescan^TM^ STL (*n* = 46)	22.95	1.73	0.200	0.031 *
	Plaster cast (*n* = 46)	23.31	2.02	0.095	0.009 *
	iTero^TM^-printed cast (*n* = 45)	22.92	1.65	0.095	0.004 *
	Primescan^TM^-printed cast (*n* = 47)	22.99	1.91	0.200	0.002 *
Canine–molar distance (left) (CMLU) (*n* = 44)	Intraoral (*n* = 46)	22.48	1.32	0.200	0.057
	iTero^TM^ STL (*n* = 45)	22.75	1.47	0.200	0.870
	Primescan^TM^ STL (*n* = 47)	22.64	1.41	0.200	0.558
	Plaster cast STL (*n* = 46)	23.81	5.18	0.000 *	0.000 *
	iTero^TM^-printed cast (*n* = 47)	22.71	1.38	0.200	0.686
	Primescan^TM^-printed cast (*n* = 48)	23.04	3.73	<0.001 *	<0.001 *

^a^ SD. Standard deviation. ^b^ Kolmogorov–Smirnov test with Lilliefors significance correction. * Statistical significance *p* ≤ 0.05.

**Table 2:** Measurements (mean and SD) and normality tests in the lower arch.

Lower Arch		Descriptive		Normality Tests	
		Mean (mm)	SD ^a^	Kolmogorov–Smirnov ^b^ *p* Value	Shapiro–Wilk *p* Value
Intercanine distance (CCL) (*n* = 42)	Intraoral (*n* = 48)	26.53	2.24	0.200	0.306
	iTero^TM^ STL (*n* = 43)	26.51	2.07	0.200	0.654
	Primescan^TM^ STL (*n* = 47)	26.34	2.25	0.200	0.291
	Plaster cast (*n* = 48)	26.45	2.11	0.200	0.967
	iTero^TM^-printed cast (*n* = 46)	26.49	2.14	0.200	0.281
	Primescan^TM^-printed cast (*n* = 46)	26.38	2.21	0.200	0.786
Intermolar distance (MML) (*n* = 48)	Intraoral (*n* = 51)	44.78	3.21	0.200	0.630
	iTero^TM^ STL (*n* = 49)	44.06	3.14	0.200	0.612
	Primescan^TM^ STL (*n* = 51)	43.98	3.17	0.200	0.424
	Plaster cast (*n* = 51)	43.77	5.25	0.000 *	0.000 *
	iTero^TM^-printed cast (*n* = 51)	44.23	3.02	0.200	0.948
	Primescan^TM^-printed cast (*n* = 50)	44.20	3.15	0.200	0.523
Canine–molar distance (right) (CMRL) (*n* = 44)	Intraoral (*n* = 48)	21.40	1.92	0.200	0.136
	iTero^TM^ STL (*n* = 44)	21.89	1.79	0.040 *	0.023 *
	Primescan^TM^ STL (*n* = 47)	21.69	2.01	0.054	0.003 *
	Plaster cast (*n* = 48)	21.97	1.91	0.108	0.024 *
	iTero^TM^-printed cast (*n* = 47)	21.85	1.80	0.182	0.013 *
	Primescan^TM^-printed cast (*n* = 47)	21.90	1.89	0.074	0.002*
Canine–molar distance (left) (CMLL) (*n* = 44)	Intraoral (*n* = 49)	21.62	1.97	0.183	0.003 *
	iTero^TM^ STL (*n* = 46)	22.36	1.61	0.081	0.054
	Primescan^TM^ STL (*n* = 50)	21.72	3.43	0.000 *	0.000 *
	Plaster cast (*n* = 49)	22.29	1.98	0.024 *	0.000
	iTero^TM^-printed cast (*n* = 49)	22.08	1.94	0.200	<0.001 *
	Primescan^TM^-printed cast (*n* = 50)	22.06	1.94	0.171	<0.001 *

^a^ SD. Standard deviation. ^b^ Kolmogorov–Smirnov test with the Lilliefors significance correction. * Statistical significance *p* ≤ 0.05.

**Table 3:** Pearson correlation coefficient for all reproduction methods in comparison to intraoral measurements.

	CCU	MMU	CMRU	CMLU	CCL	MML	CMRL	CMLL
Intraoral*iTero^TM^ STL	0.921	0.869	0.828	0.824	0.954	0.956	0.797	0.820
Intraoral*Primescan^TM^ STL	0.895	0.979	0.811	0.867	0.957	0.966	0.855	0.509
Intraoral*Plaster cast	0.934	0.970	0.701	0.255	0.758	0.376	0.830	0.824
Intraoral*iTero^TM^-printed cast	0.908	0.898	0.830	0.783	0.952	0.953	0.817	0.858
Intraoral*Primescan^TM^-printed cast	0.888	0.962	0.811	0.889	0.971	0.966	0.849	0.859

* Pearson coefficient correlation interpreted as a very low correlation (0 < r ≤ 0.19), low correlation (0.2 ≤ r ≤ 0.39), moderate correlation (0.4 ≤ r ≤ 0.59), high correlation (0.6 ≤ r ≤ 0.79) or very high correlation (0.8 ≤ r ≤ 1).

**Table 4:** Significance of paired tests in the upper arch.

		CCU ^a^	MMU ^a^	CMRU ^b^	CMLU ^b^
**Comparison 1**	Intraoral	0.028 *	0.292	0.204	0.210
	iTero^TM^ STL				
**Comparison 2**	Intraoral	0.044 *	<0.001 *	0.726	0.346
	Primescan^TM^ STL				
**Comparison 3**	Intraoral	0.163	0.590	0.051	0.005 *
	Plaster cast				
**Comparison 4**	Intraoral	0.712	0.546	0.942	0.250
	iTero^TM^-printed cast				
**Comparison 5**	Intraoral	0.190	0.015 *	0.636	0.844
	Primescan^TM^-printed cast				

^a^ *t*-test. ^b^ Wilcoxon rank test. * Statistical significance *p* ≤ 0.05.

**Table 5:** Significant paired tests in the lower arch.

		CCL ^a^	MML ^b^	CMRL ^b^	CMLL ^b^
**Comparison 1**	Intraoral	0.382	<0.001 *	0.071	0.001 *
	iTero^TM^ STL				
**Comparison 2**	Intraoral	0.082	<0.001 *	0.110	0.031 *
	Primescan^TM^ STL				
**Comparison 3**	Intraoral	0.719	0.619	0.002 *	<0.001 *
	Plaster cast				
**Comparison 4**	Intraoral	0.871	<0.001 *	0.003 *	0.036 *
	iTero^TM^-printed cast				
**Comparison 5**	Intraoral	0.268	<0.001 *	0.004 *	0.033 *
	Primescan^TM^-printed cast				

^a^ *t*-test. ^b^ Wilcoxon rank test. * Statistical significance *p* ≤ 0.05.

## Data Availability

The raw data presented in this study are available on request from the corresponding author (A.M.-V.) due to privacy restrictions.

## Appendix A

**Table A1:** Technical specifications of the electronic digital caliper (Vietnam E-Commerce Limited, RM 18 27/F, Ho King, COMM CTR 2 16 FA Yueng St. Mongkok, Kowloon, Hong Kong) according to the manufacturer.

**Measure range**	0–75 mm/0–3″
	0–100 mm/0.4″
	0–150 mm/0.6″
	0–200 mm/0.8″
	0–300 mm/0.12″
**Resolution**	0.01 mm/0.0005″
**Accuracy**	±0.02 mm/0.001″ (<100 mm)
	±0.03 mm/0.001″ (>100–200 mm)
	±0.04 mm/0.0015″ (>200–300 mm)
**Repeatability**	0.01 mm/0.0005″
**Measuring system**	Linear capacitive measuring system
**Working temperature**	5~40 °C/41~104 °C
**Influence of humidity**	Not important under 80% of relative humidity
